# Supplementary material for: An open-source framework for large-scale, flexible evaluation of biomedical text mining systems
Source: J Biomed Discov Collab. 2008 Jan 29;3:1. doi: 10.1186/1747-5333-3-1 (PMC2276192; doi:10.1186/1747-5333-3-1)
Supplement: Additional file 1 — Evaluation platform source code. The additional file contains the software evaluation framework discussed in this paper. The most current release can be downloaded from SourceForge.net [1]. See the accompanying README file for instructions on installation and use. Also included in the distribution is a collection of UIMA wrappers for some commonly used BioNLP tools and annotated corpora. [file 1747-5333-3-1-S1.zip › bionlp_uima_repository-1.0.1/docs/api/src-html/edu/uchsc/ccp/uima/annotation/syntactic/CCPPhraseAnnotation_Type.html]

```
001    
002    /* First created by JCasGen Tue Aug 28 16:44:10 MDT 2007 */
003    package edu.uchsc.ccp.uima.annotation.syntactic;
004    
005    import org.apache.uima.cas.Feature;
006    import org.apache.uima.cas.FeatureStructure;
007    import org.apache.uima.cas.Type;
008    import org.apache.uima.cas.impl.CASImpl;
009    import org.apache.uima.cas.impl.FSGenerator;
010    import org.apache.uima.cas.impl.FeatureImpl;
011    import org.apache.uima.cas.impl.TypeImpl;
012    import org.apache.uima.jcas.JCas;
013    import org.apache.uima.jcas.JCasRegistry;
014    
015    import edu.uchsc.ccp.uima.annotation.CCPSyntacticAnnotation_Type;
016    
017    /** This annotation type is used to annotation phrases.
018     * Updated by JCasGen Tue Aug 28 16:44:10 MDT 2007
019     * @generated */
020    public class CCPPhraseAnnotation_Type extends CCPSyntacticAnnotation_Type {
021      /** @generated */
022      protected FSGenerator getFSGenerator() {return fsGenerator;}
023      /** @generated */
024      private final FSGenerator fsGenerator = 
025        new FSGenerator() {
026          public FeatureStructure createFS(int addr, CASImpl cas) {
027                             if (CCPPhraseAnnotation_Type.this.useExistingInstance) {
028                               // Return eq fs instance if already created
029                         FeatureStructure fs = CCPPhraseAnnotation_Type.this.jcas.getJfsFromCaddr(addr);
030                         if (null == fs) {
031                           fs = new CCPPhraseAnnotation(addr, CCPPhraseAnnotation_Type.this);
032                               CCPPhraseAnnotation_Type.this.jcas.putJfsFromCaddr(addr, fs);
033                               return fs;
034                         }
035                         return fs;
036            } else return new CCPPhraseAnnotation(addr, CCPPhraseAnnotation_Type.this);
037              }
038        };
039      /** @generated */
040      public final static int typeIndexID = CCPPhraseAnnotation.typeIndexID;
041      /** @generated 
042         @modifiable */
043      public final static boolean featOkTst = JCasRegistry.getFeatOkTst("edu.uchsc.ccp.uima.annotation.syntactic.CCPPhraseAnnotation");
044     
045      /** @generated */
046      final Feature casFeat_phraseType;
047      /** @generated */
048      final int     casFeatCode_phraseType;
049      /** @generated */ 
050      public String getPhraseType(int addr) {
051            if (featOkTst && casFeat_phraseType == null)
052          jcas.throwFeatMissing("phraseType", "edu.uchsc.ccp.uima.annotation.syntactic.CCPPhraseAnnotation");
053        return ll_cas.ll_getStringValue(addr, casFeatCode_phraseType);
054      }
055      /** @generated */    
056      public void setPhraseType(int addr, String v) {
057            if (featOkTst && casFeat_phraseType == null)
058          jcas.throwFeatMissing("phraseType", "edu.uchsc.ccp.uima.annotation.syntactic.CCPPhraseAnnotation");
059        ll_cas.ll_setStringValue(addr, casFeatCode_phraseType, v);}
060        
061      
062    
063    
064    
065      /** initialize variables to correspond with Cas Type and Features
066            * @generated */
067      public CCPPhraseAnnotation_Type(JCas jcas, Type casType) {
068        super(jcas, casType);
069        casImpl.getFSClassRegistry().addGeneratorForType((TypeImpl)this.casType, getFSGenerator());
070    
071     
072        casFeat_phraseType = jcas.getRequiredFeatureDE(casType, "phraseType", "uima.cas.String", featOkTst);
073        casFeatCode_phraseType  = (null == casFeat_phraseType) ? JCas.INVALID_FEATURE_CODE : ((FeatureImpl)casFeat_phraseType).getCode();
074    
075      }
076    }
077    
078    
079    
080
```
